# Hippocampal, thalamic, and amygdala subfield morphology in major depressive disorder: an ultra-high resolution MRI study at 7-Tesla

**DOI:** 10.1007/s00406-024-01874-0

**Published:** 2024-08-31

**Authors:** Weijian Liu, Jurjen Heij, Shu Liu, Luka Liebrand, Matthan Caan, Wietske van der Zwaag, Dick J. Veltman, Lin Lu, Moji Aghajani, Guido van Wingen

**Affiliations:** 1 Peking University Sixth Hospital, Peking University Institute of Mental Health, NHC Key Laboratory of Mental Health (Peking University), National Clinical Research Center for Mental Disorders (Peking University Sixth Hospital), Peking University, HuayuanBei Road 51, Beijing, 100191 China; 2 Department of Psychiatry, UMC Location University of Amsterdam, Meibergdreef 5, 1100 DD Amsterdam, the Netherlands; 3 Amsterdam Neuroscience, Amsterdam, the Netherlands; 4 Spinoza Centre for Neuroimaging, KNAW, Amsterdam, the Netherlands; 5 Department of Computational Cognitive Neuroscience and Neuroimaging, Netherlands Institute for Neuroscience, Amsterdam, Netherlands; 6 Key Laboratory of Genetic Evolution & Animal Models, National Research Facility for Phenotypic & Genetic Analysis of Model Animals (Primate Facility), National Resource Center for Non-Human Primates, Kunming Institute of Zoology, Chinese Academy of Sciences, Kunming, China; 7 Department of Radiation Oncology, Amsterdam UMC Location Vrije Universiteit Amsterdam, Amsterdam, the Netherlands; 8 Department of Biomedical Engineering & Physics, UMC Location University of Amsterdam, Amsterdam, the Netherlands; 9 Department of Psychiatry, Amsterdam UMC Location Vrije Universiteit Amsterdam, Amsterdam, Netherlands; 10 Peking-Tsinghua Centre for Life Sciences and PKU-IDG/McGovern Institute for Brain Research, Peking University, Beijing, China; 11 National Institute On Drug Dependence and Beijing Key Laboratory of Drug Dependence, Peking University, Beijing, China; 12 Institute of Education and Child Studies, Section Forensic Family and Youth Care, Leiden University, Leiden, the Netherlands

**Keywords:** Ultra-high field MRI, 7.0 Tesla, Hippocampus, Thalamus, Amygdala, Major depressive disorder

## Abstract

Morphological changes in the hippocampal, thalamic, and amygdala subfields have been suggested to form part of the pathophysiology of major depressive disorder (MDD). However, the use of conventional MRI scanners and acquisition techniques has prevented in-depth examinations at the subfield level, precluding a fine-grained understanding of these subfields and their involvement in MDD pathophysiology. We uniquely employed ultra-high field MRI at 7.0 Tesla to map hippocampal, thalamic, and amygdala subfields in MDD. Fifty-six MDD patients and 14 healthy controls (HCs) were enrolled in the final analysis. FreeSurfer protocols were used to segment hippocampal, thalamic, and amygdala subfields. Bayesian analysis was then implemented to assess differences between groups and relations with clinical features. While no effect was found for MDD diagnosis (i.e., case–control comparison), clinical characteristics of MDD patients were associated with subfield volumes of the hippocampus, thalamus, and amygdala. Specifically, the severity of depressive symptoms, insomnia, and childhood trauma in MDD patients related to lower thalamic subfield volumes. In addition, MDD patients with typical MDD versus those with atypical MDD showed lower hippocampal, thalamic, and amygdala subfield volumes. MDD patients with recurrent MDD versus those with first-episode MDD also showed lower thalamic subfield volumes. These findings allow uniquely fine-grained insights into hippocampal, thalamic, and amygdala subfield morphology in MDD, linking some of them to the clinical manifestation of MDD.

**Supplementary Information:**

The online version contains supplementary material available at 10.1007/s00406-024-01874-0.

## Introduction

Major depressive disorder (MDD) poses a significant burden to society [1, 2]. Although MDD is increasingly being studied around the world, its pathophysiological mechanisms remain elusive [3, 4]. Various studies have shown that patients with MDD have decreased grey matter volume in certain brain regions [3]. The most common is a volumetric reduction in the hippocampus, a region that is crucial for learning, memory, and emotions [5]. Two large meta-analyses showed that patients with MDD have smaller hippocampal volumes than healthy controls (HCs) [6, 7], though many studies have not been able to detect the subtle differences [8, 9]. Located just in front of and intricately intertwined with the hippocampus is the amygdala, which has a crucial role in the affective/mood states and processing of emotions [10–12]. Research on differences in amygdala volume between patients with depression and HCs has been inconsistent [10]. Studies showed smaller [13, 14], larger [15], and unchanged [16] amygdala volumes in patients with depression relative to HCs. Another research hotspot is the thalamus, a key node within various emotion processing networks [17, 18], deemed to play an important role in the pathophysiology of MDD [19]. Most studies showed that patients with MDD have significantly reduced overall volumes of the thalamus, either bilaterally or on the left side, compared with HCs [6, 20–22]. A study also showed that reduced thalamic gray matter volumes correlate with the severity of depressive symptoms [21]. However, there is also evidence that patients with MDD have increased or no difference at all in thalamic volumes when compared with HCs [23–25].

The hippocampus, thalamus, and amygdala are composed of functionally and structurally segregated subfields, which are tentatively implicated to underpin different pathophysiological mechanisms in depression [19, 26, 27]. Pieces of evidence indicate that hippocampal involvement in MDD pathology is limited to only some of its subfields. A study showed that the volume of the bilateral hippocampal tail and the right hippocampal head were smaller than those in HCs [28]. Another study indicated that patients with MDD who were in remission after taking psychotropic medication for 8 weeks had larger bilateral hippocampal body/tail volumes before treatment than those who were not in remission [29]. Yet another study indicated that the right amygdala medial nucleus volume was smaller in HC compared to MDD [10]. Regarding thalamic subnuclei, MDD patients were found to have smaller volumes than HCs in several left thalamic nuclei, including lateral, ventral, intralaminar, medial, and posterior regions [30]. These studies, however, were all conducted using standard field strength MRI scanners (1.5/3.0 Tesla) with conventional acquisition techniques, thus precluding thorough and precise examinations at the subfield level.

The use of ultra-high field (UHF) MRI at 7.0 Tesla and above is increasingly postulated to overcome these limitations, as the increased signal-to-noise ratio at UHF may be used to greatly enhance spatial resolution it greatly enhances spatial resolution [31–33], thereby enabling improved and more precise segmentation of small deep-lying brain structures [34]. Due to technical and financial limitations, very few studies have used UHF MRI to explore the structural integrity of hippocampal, thalamic, and amygdala subfields in MDD. One study indicated that remitted MDD patients had larger volumes of the right fissure and the right hippocampal-amygdala transition area (HATA) compared with HCs [35]. However, another study interestingly revealed that while MDD diagnosis does not relate to hippocampal subfield changes, the number of depressive episodes does correlate with decreased subiculum subfield volumes [36]. Another study similarly found no differences in the volumes of amygdala subfield in MDD, yet did observe a negative correlation between subfield volumes (right lateral nucleus (La), left cortical nucleus (Co), left accessory basal nucleus (AB) and cortico-amygdaloid transition area (CAT)) and the severity of depressive symptoms [37].

The limited UHF MRI evidence on structural morphology of hippocampal, thalamic, and amygdala subfields in MDD warrants further validation and exploration, in order to gain a more fine-grained and microscale understanding of its pathophysiology. We therefore used the power of UHF MRI at 7.0 Tesla to further explore hippocampal, thalamic, and amygdala subfield volumes in MDD. Based on prior work on the topic [5, 38, 39], effects were hypothesized to occur in emotion-related (CA1, CAT, HATA, thalamic posterior nuclei), anxiety-related (thalamic medial nuclei, AB), and stress-related (CA3) subnuclei of the thalamus, hippocampus, and amygdala. We additionally explored whether clinical variables influenced subfield volumetrics in MDD patients.

## Method

### Participant recruitment

Patients with MDD recruited in this study met the following inclusion criteria: (1) a primary diagnosis of current MDD that occurred within the last 6 months, according to DSM-5 criteria, as determined by the Composite International Diagnostic Interview (CIDI) [40]; (2) aged between 20–55. HCs met the following inclusion criteria: (1) no history of depression diagnosis or treatment, nor any other psychopathology; (2) normal or subclinical scores on dimensional measures of psychopathology; (3) aged between 20–55. Exclusion criteria for the entire sample: (1) presence of psychoses, mania, Tourette’s syndrome, or obsessive–compulsive disorder; (2) diagnosis of major internal or neurological disorders; (3) traumatic head injury; (4) current substance abuse or dependence requiring treatment; (5) evidence of acute suicidal risk requiring immediate intervention; (6) MRI contradictions, including metal implants, heart arrhythmia, or claustrophobia; (7) left-handedness; (8) pregnancy; (9) inadequate understanding of the Dutch language. After excluding one MDD patient with poor T1-weighted image quality, a total of 56 MDD participants and 14 HCs were included in the final analysis. Due to the financial burden and time constraints at play when conducting UHF MRI research, we chose to maximize and prioritize the inclusion of our main target group (MDD), which resulted in a relatively small HC sample. That said, the 56 MDD patients included here render this the largest UHF structural MRI examination of the hippocampal, thalamic, and amygdala subfield morphology in MDD to date. Detailed demographic and clinical information are shown in Table 1. The ethical review board of the Amsterdam UMC (location VUmc) approved this study, and written informed consent was obtained from all participants.

**Table 1:** Demographical and clincal information of the participants

	MDD (n = 56)	HC (n = 14)	BF_10_	Strength of evidence
Age (years), mean ± SD	36.54 ± 10.53	35.18 ± 9.45	0.319	None
Gender (female), n (%)	42 (75.00%)	8 (57.14%)	0.616	None
Age of onset (years) ^a^, mean ± SD	21.53 ± 10.72	–	–	–
Recurrent depression, n (%)	23 (41.07%)	–	–	–
With atypical MDD ^a^, n (%)	14 (25.00%)	–	–	–
IDS ^a^, mean ± SD	34.06 ± 13.45	3.93 ± 2.87	**7.152E8**	**Extreme**
CTQ ^a^, mean ± SD	47.27 ± 17.61	38.00 ± 8.77	1.257	None
BAI ^a^, mean ± SD	14.13 ± 9.58	2.14 ± 2.28	**935.291**	**Extreme**
IRS ^a^, mean ± SD	10.02 ± 5.21	5.29 ± 4.30	**14.038**	**Strong**
With any psychotropic medications, n (%)	32 (57.14%)	–	–	–
With any antidepressants, n (%)	29 (51.79%)	–	–	–
With SSRIs and/or SNRIs, n (%)	22 (39.29%)	–	–	–
With TCA, n (%)	8 (14.29%)	–	–	–
With atypical antidepressants, n (%)	2 (3.57%)	–	–	–
With lithium, n (%)	3 (5.26%)	–	–	–
With antipsychotics, n (%)	6 (10.71%)	–	–	–
With benzodiazepines, n (%)	7 (12.50%)	–	–	–

Bold indicates moderate or greater evidence

*MDD* major depressive disorder, *HC* healthy control, *SD* standard deviation, *IDS* Inventory for Depressive Symptomatology, *CTQ* Childhood Trauma Questionnaire, *BAI* Becks Anxiety Inventory, *IRS* Insomnia Rating Scale, *SSRI* selective serotonin reuptake inhibitor, *SNRI* serotonin-norepinephrine reuptake inhibitor, *TCA* tricyclic antidepressant

^a^ One MDD patient did not provide the age of onset, and another MDD patient did not provide data for IDS, BAI, IRS, and CTQ. Therefore, when analyzing the data related to these indicators, the sample size for MDD is 55 individuals

### Clinical interview and assessment

Clinical interviews and assessments were finished on the same day of MRI scanning. The Inventory for Depressive Symptomatology (IDS) [41], the Becks Anxiety Inventory (BAI) [42], the Insomnia Rating Scale (IRS) [43], and the Childhood Trauma Questionnaire (CTQ) [44] were used to measure the severity of depression, anxiety, insomnia, and childhood trauma, respectively. In line with prior work, we also used the IDS to subtype MDD patients in “typical” and “atypical” subtypes. To meet the criteria for atypical MDD, patients needed to have specific scores on various IDS items. Emotional reactivity required scores of 0, 1, or 2, leaden paralysis required scores of 2 or 3, weight gain or increased appetite required scores of 2 or 3, hypersomnia required scores of 2 or 3, and interpersonal sensitivity required a score of 3. Furthermore, to be classified as having atypical MDD, patients had to be emotionally reactive (not scoring 3 on the emotional response item) and meet the criteria for at least 2 of the other 4 symptoms [45].

### MRI acquisition

Images were acquired using a Philips Achieva 7T MRI scanner with a 32-channel head array coil. T1 weighted images were obtained as anatomical reference using an MP2RAGEME (multi-echo magnetization-prepared rapid gradient echo) sequence [46]. The MP2RAGEME is an extension of the MP2RAGE sequence [47] and consists of two rapid gradient echo (GRE_1,2_) images that are acquired after a 180° degrees inversion pulse and excitation pulses with inversion times TI_1,2_ = [670 ms, 3675.4 ms]. A multi-echo readout was used in the second inversion, with four equally spaced echo times (TE_1_ = 3 ms, TE_2,1–4_ = 3, 11.5, 19, 28.5 ms). Other scan parameters include flip angles_1,2_ = [4°, 4°]; TR_GRE1,2_ = [6.2 ms, 31 ms]; bandwidth = 404.9 MHz; TR_MP2RAGEME_ = 6778 ms; acceleration factor SENSE_PA_ = 2; FOV = 205 × 205 × 164 mm; acquired voxel size = 0.7 × 0.7 × 0.7 mm; acquisition matrix was 292 × 290; reconstructed voxel size 0.64 × 0.64 × 0.7 mm; turbo factor (TFE) = 150, resulting in 176 shots; Total acquisition time = 19.53 min.

### Subfields segmentation of the hippocampus, thalamus, and amygdala

The T1-weighted images were preprocessed using the “recon-all” pipeline with FreeSurfer version 7.2.0 (http://surfer.nmr.mgh.harvard.edu). The preprocessing steps include normalization of signal intensity, skull stripping, talairach correction, and automated segmentation of subcortical white matter and grey matter structures [48].

**Fig. 1:**
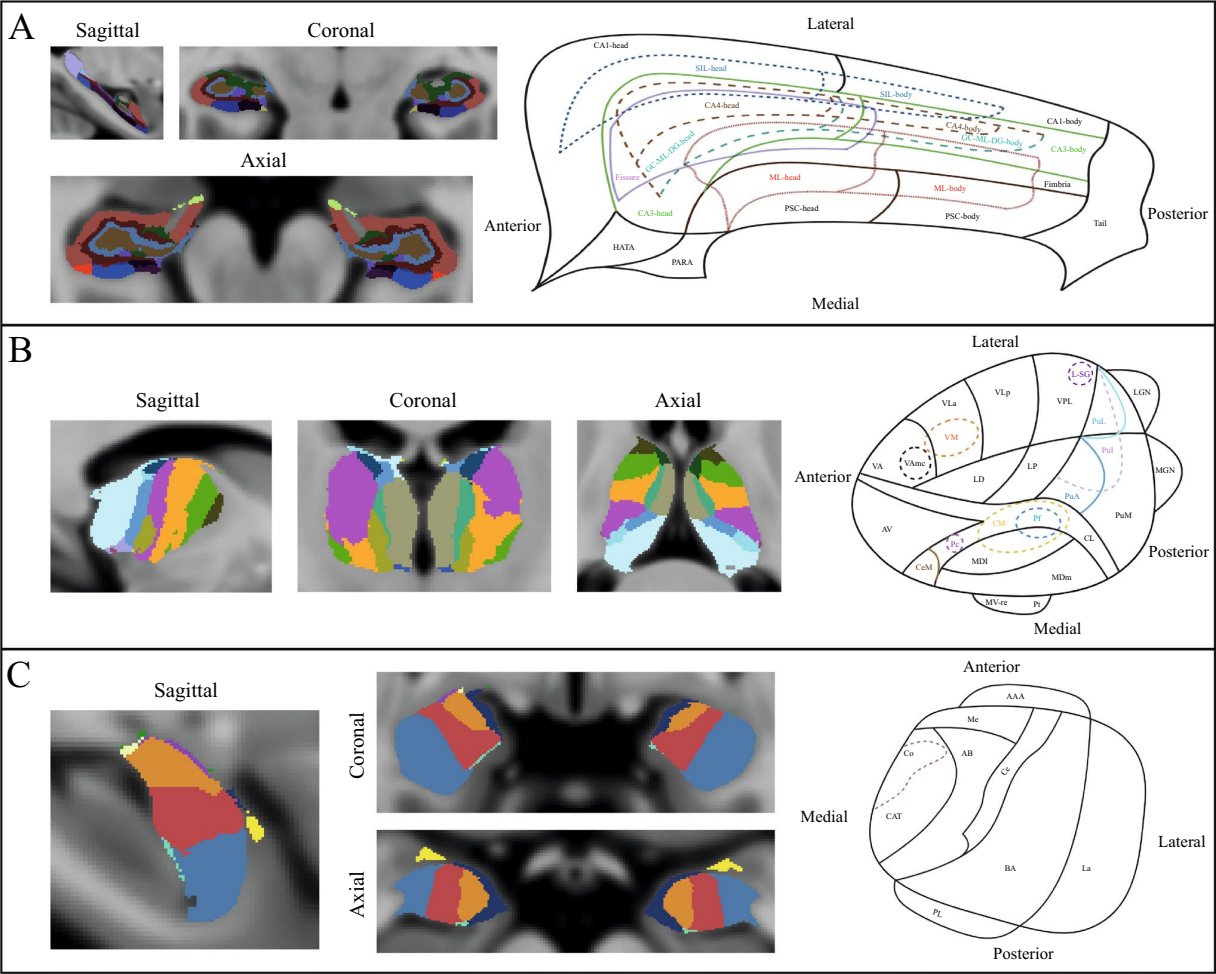
Subfields of hippocampus, thalamus, and amygdala. (**A**) hippocampal subfields; (**B**) thalamic subfields; (**C**) amygdala subfields. *PARA* parasubiculum, *PSIL* presubiculum, *SIL* subiculum, *CA* cornu Ammonis, *GC-ML-DG* granule cell and molecular layer of the dentate gyrus, *ML* molecular layer, *HATA* hippocampal-amygdala transition area, *AV* anteroventral nucleus, *LD* laterodorsal nucleus, *LP* lateral posterior nucleus, *VA* ventral anterior nucleus, *VAmc* ventral anterior magnocellular nucleus, *Vla* ventral lateral anterior nucleus, *VLp* ventral lateral posterior nucleus, *VPL* ventral posterolateral nucleus, *VM* ventromedial nucleus, *CM* centromedian nucleus, *CeM* central medial nucleus, *CL* central lateral nucleus, *Pc* paracentral nucleus, *Pf* parafascicular nucleus, *Pt* paratenial nucleus, *MV-re* medial ventral reuniens nucleus, *MDm* mediodorsal medial magnocellular nucleus, *MDI* mediodorsal lateral parvocellular nucleus, *LGN* lateral geniculate nucleus, *MGN* medical geniculate nucleus, *L-SG* limitans-suprageniculate nucleus, *PuA* pulvinar anterior nucleus, *PuM* pulvinar medial nucleus, *PuL* pulvinar lateral nucleus, *PuI* pulvinar inferior nucleus, *La* lateral nucleus, *Ba* basal nucleus, *AB* accessory basal nucleus, *Ce* central nucleus, *Me* medial nucleus, *Co* cortical nucleus, *PL* paralaminar nucleus, *CAT* cortico-amngdaloid transition area; *AAA* anterior amygdala area

Afterwards, the preprocessed T1-weighted images were subjected to thalamic, hippocampal, and amygdala segmentation using FreeSurfer’s subfield segmentation module, which demonstrated excellent test–retest reliability and remained robust to variations in input MRI contrast [19, 26, 27]. In this step, as it is showed in Fig. 1, (1) the thalamus was segmented into 25 different subfields: anteroventral (AV), laterodorsal (LD), lateral posterior (LP), ventral anterior (VA), ventral anterior magnocellular (VAmc), ventral lateral anterior (VLa), ventral lateral posterior (VLp), ventral posterolateral (VPL), ventromedial (VM), centromedian (CM), central medial (CeM), central lateral (CL), paracentral (Pc), parafascicular (Pf), paratenial (Pt), medial ventral reuniens (MV-re), mediodorsal medial magnocellular (MDm), mediodorsal lateral parvocellular (MDI), lateral geniculate (LGN), medical geniculate (MGN), limitans-suprageniculate (L-SG), pulvinar anterior (PuA), pulvinar medial (PuM), pulvinar lateral (PuL), and pulvinar inferior (PuI) nuclei; (2) the hippocampus was segmented to 19 subfields: parasubiculum (PARA), presubiculum (PSIL)-head/body, subiculum (SIL)-head/body, cornu Ammonis 1 (CA1)-head/body, CA3-head/body, CA4-head/body, granule cell and molecular layer of the dentate gyrus (GC-ML-DG)-head/body, molecular layer (ML)-head/body, HATA, fimbria, tail, and fissure; (3) and the amygdala was segmented into 9 subfields: La, basal (Ba), AB, central (Ce), medial (Me), Co and paralaminar (PL) nuclei, and CAT and anterior amygdala areas (AAA). Segmentations were inspected manually. Volumetrics of each subfield are computed upon a soft segmentation, and extracted for further analyses.

### Statistical analysis

We conducted our statistical analyses using Bayesian statistics as implemented in JASP (version 0.17.1: https://jasp-stats.org/) [49]. An important advantage of Bayesian statistics is that it can provide evidence for the alternative as well as the null hypothesis. Moreover, Bayesian statistics render multiple comparisons correction redundant, as the posterior distribution does not change when additional comparisons are made [50]. The Bayesian factor (BF) was interpreted using the following evidence categories: BF < 3 (and its reciprocal) indicates anecdotal evidence for Hypothesis 1; BF ≥ 3 corresponds to moderate evidence; BF ≥ 10 suggests strong evidence; BF ≥ 30 represents very strong evidence; and BF ≥ 100 indicates extreme evidence [51]. Results with BF < 3 were considered to present insufficient evidence, and only results with BF ≥ 3 are reported and discussed.

For basic demographic and clinical data comparisons, the Bayesian chi-square test was used for dichotomous variables, and the Bayesian independent samples t-test for continuous variables. A Bayesian analysis of covariance (ANCOVA) was performed on every subfield, including group (MDD/HC) or subgroups (medicated/unmedicated MDD; recurrent episodes/first episode MDD; typical/atypical MDD) as a fixed factor. To assess whether a clinical subgroup also differed from HC, post-hoc Bayesian ANCOVAs were conducted to compare every subfield volume between subgroups and HC (medicated MDD/HC, unmedicated MDD/HC, recurrent episodes MDD/HC, first episode MDD/HC, typical MDD/HC, atypical MDD/HC). Age, gender, and intracranial volume (ICV) were included as covariates in all ANCOVAs. Bayesian ANCOVA works by comparing four models with varying predictors of final course grade: (1) a null model; (2) a model containing only the group as a predictor; (3) models containing only one covariate; (4) several models containing interaction between fixed factor and covariates as predictors. Then we obtained the BF_M_, which indicated the change in model odds after observing the data. To account for model uncertainty, we then performed Bayesian model averaging to test the effect of the predictors to obtain BF_incl_, a primary measure of the strength of evidence for differences between groups.

Finally, Bayesian linear regression analysis was performed to examine if the severity of depression, anxiety symptoms, insomnia, childhood trauma, and age of onset were associated with volumes of subfields among MDD patients. Age, gender, and ICV were again used as covariates, with subfields as dependent variables. For highly skewed clinical data, we applied a square root transformation to normalize the data. In the Bayesian linear regression model, we applied a uniform prior to the models, such that all models were set to be equally likely a priori before observing any data, and we applied Jeffreys-Zellner-Siow (JZS) prior (r scale = 0.354) to the regression coefficients. Results were only retained if there was at least moderate evidence (BF ≥ 3) after the removal of outliers.

## Results

### Demographical and clinical variables

A total of 56 MDD patients (age = 36.54 ± 10.53 years) and 14 HCs (age = 35.18 ± 9.45 years) were enrolled in the present study. Among the MDD patients, 23 individuals had recurrent depression (41%), 14 individuals had typical depression (25%), with a mean age of onset of 21.53 ± 10.72 years. For the medication status, 32 (57%) of MDD patients were medicated, 29 (52%) were on antidepressants, 3 (5%) were on lithium, 6 (11%) were on antipsychotics, and 7 (13%) were on benzodiazepines. The Bayesian independent samples t-test and the Bayesian chi-square test provided no evidence that age, gender, and scores of CTQ were different between groups (all BF_10_ < 3). Moderate or greater evidence was found for MDD patients having higher scores of IDS, BAI, and IRS (all BF_10_ > 3). Demographical and clinical details are shown in Table 1.

### Differences in subfield volumes between MDD and HC

The Bayesian ANCOVAs showed no evidence for any conclusive differences in hippocampal, thalamic, or amygdala subfield nor whole volumes in MDD versus HC (Supplemental Table 1–3).

### Differences in subfield volumes between MDD subgroups

**Fig. 2:**
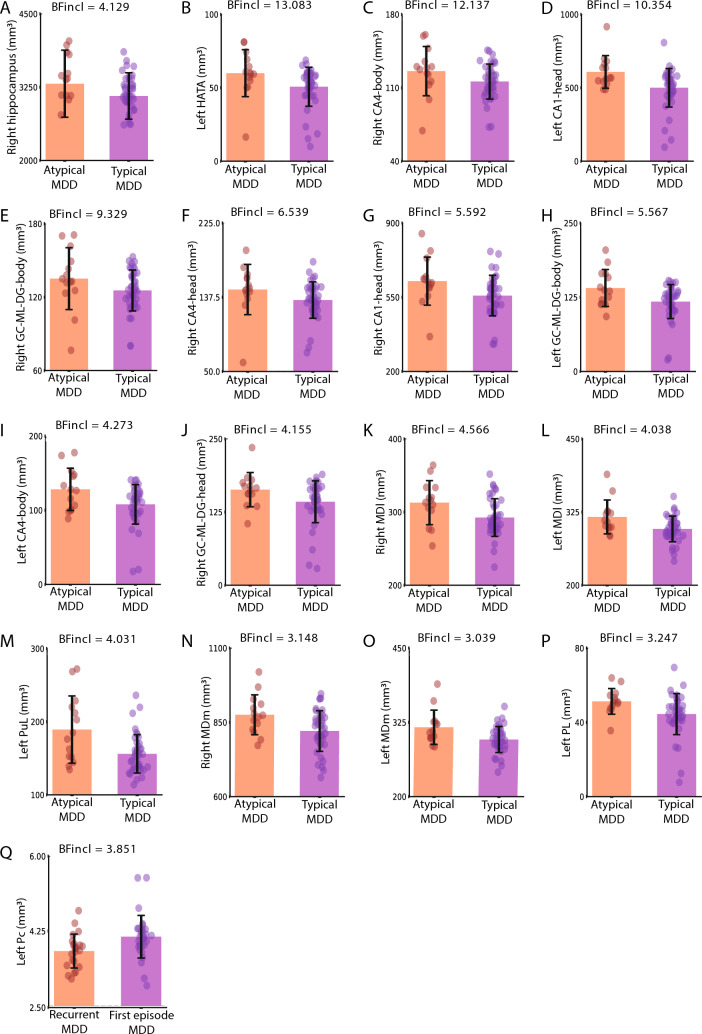
Differences in volumes of the hippocampal, thalamic, and amygdala subfields between groups. *MDD* major depressive disorder, *HATA* hippocampal-amygdala transition area, *CA* cornu Ammonis, *GC-ML-DG* granule cell and molecular layer of the dentate gyrus, *MDl* mediodorsal lateral parvocellular, *PuL* pulvinar lateral, *MDm* mediodorsal medial magnocellular, *PL* paralaminar nuclear, *Pc* paracentral nuclear

While separating the MDD group into typical and atypical MDD, Bayesian ANCOVA provided moderate to strong evidence for patients with typical MDD having lower volumes within right whole hippocampus (BF_incl_ = 4.129, Table 2, Fig. 2. A), nine hippocampal subfields (left HATA: BF_incl_ = 13.083, right CA4-body: BF_incl_ = 12.137, left CA1-head: BF_incl_ = 10.354, right GC-ML-DG-body: BF_incl_ = 9.329, right CA4-head: BF_incl_ = 6.539, right CA1-head: BF_incl_ = 5.592, left GC-ML-DG-body: BF_incl_ = 5.567, left CA4-body: BF_incl_ = 4.273, and right GC-ML-DG-head: BF_incl_ = 4.155, Table 2, Fig. 2. B-J), five thalamic subfields (right MDl: BF_incl_ = 4.566, left MDl: BF_incl_ = 4.038, left PuL: BF_incl_ = 4.031, right MDm: BF_incl_ = 3.148, and left MDm: BF_incl_ = 3.039, Table 2, Fig. 2. K–O), and one amygdala subfield (left PL: BF_incl_ = 3.247, Table 2, Fig. 2. P). Moreover, lower volumes within one thalamic subfield (left Pc: BF_incl_ = 3.851, Table 2, Fig. 2. Q) were observed in recurrent MDD patients compared to first-episode MDD patients, with moderate evidence. Analysis for medicated versus unmedicated MDD was inconclusive (all BF_incl_ < 3).

**Table 2:** Summary of the BF values of subfield volumes for group differences

	BF_incl_	Strength of evidence
MDD versus HC		
None	< 3.000	None
Typical versus atypical MDD		
Hippocampus		
Left HATA	**13.083**	**Strong**
Right CA4-body	**12.137**	**Strong**
Left CA1-head	**10.354**	**Strong**
Right GC-ML-DG-body	**9.329**	**Moderate**
Right CA4-head	**6.539**	**Moderate**
Right CA1-head	**5.592**	**Moderate**
Left GC-ML-DG-body	**5.567**	**Moderate**
Left CA4-body	**4.273**	**Moderate**
Right GC-ML-DG-head	**4.155**	**Moderate**
Right hippocampus	**4.129**	**Moderate**
Thalamus		
Right MDl	**4.566**	**Moderate**
Left MDl	**4.038**	**Moderate**
Left PuL	**4.031**	**Moderate**
Right MDm	**3.148**	**Moderate**
Left MDm	**3.039**	**Moderate**
Amygdala		
Left PL	**3.247**	**Moderate**
First episode versus recurrent MDD		
Hippocampus		
None	< 3.000	None
Thalamus		
Left Pc	**3.851**	**Moderate**
Amygdala		
None	< 3.000	None
Medicated versus unmedicated MDD		
None	< 3.000	None

Bold indicates moderate or greater evidence

*MDD* major depressive disorder, *HC* healthy control, *HATA* hippocampal-amygdala transition area, *CA* cornu Ammonis, *GC-ML-DG* granule cell and molecular layer of the dentate gyrus, *MDl* mediodorsal lateral parvocellular, *PuL* pulvinar lateral, *MDm* mediodorsal medial magnocellular, *PL* paralaminar nuclear, *Pc* paracentral nuclear

### Differences in subfield volumes between MDD subgroups and HC

The Bayesian ANCOVA showed no evidence for any conclusive differences in hippocampal, thalamic, or amygdala subfield volumes in MDD subgroups versus HC (Supplemental Table 4).

### Relation between subfield volumes and MDD features

**Fig. 3:**
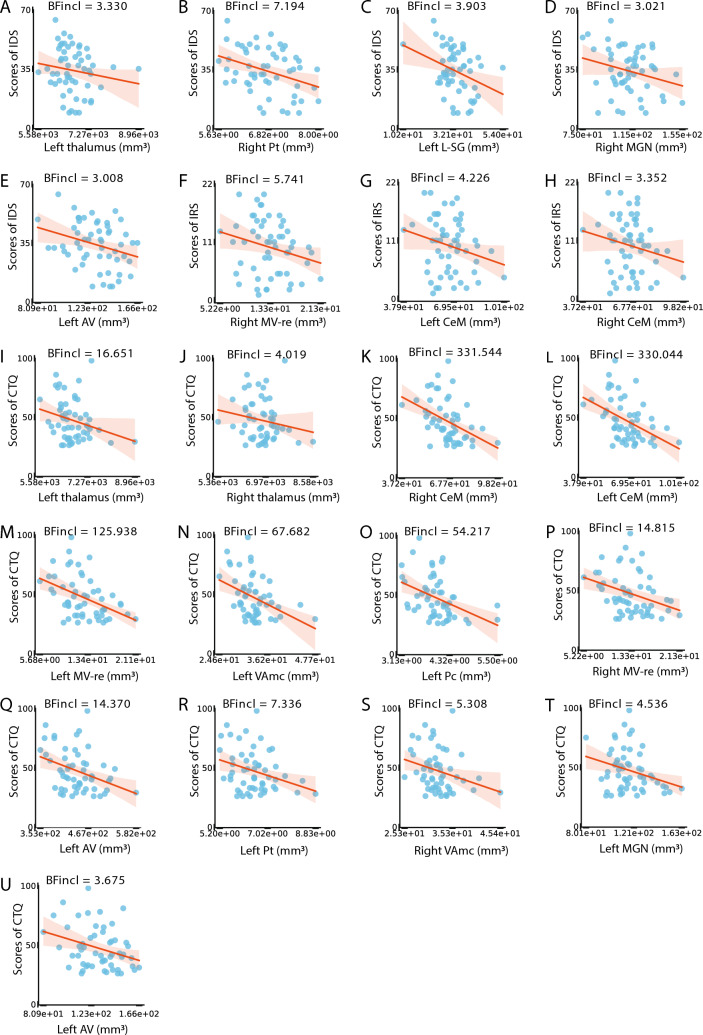
Relationships between volumes of the hippocampal, thalamic, and amygdala subfields and clinical indices in MDD. *MDD* major depressive disorder, *Pt* paratenial nuclear, *L-SG* limitans-suprageniculate nuclear, *MGN* medical geniculate nuclear, *AV* anteroventral nuclear, *MV-re* medial ventral reuniens nuclear, *CeM* central medial nuclear, *VAmc* ventral anterior magnocellular nuclear, *Pc* paracentral nuclear, *VA* ventral anterior nuclear

Moderate to strong evidence was provided that higher IDS scores related to lower volumes of left whole thalamus (BF_incl_ = 3.330, Table 3, Fig. 3. A) and four thalamic subfields (right Pt: BF_incl_ = 7.194, left L-SG: BF_incl_ = 3.903, right MGN: BF_incl_ = 3.021, and left AV: BF_incl_ = 3.008) in MDD (Table 3, Fig. 3. B-E).

**Table 3:** Summary of the BF values of subfield volumes with moderate or higher evidence for the relationship with clinical features

	BF_incl_	Strength of evidence
IDS		
Hippocampus		
None	< 3.000	None
Thalamus		
Right Pt	**7.194**	**Moderate**
Left L-SG	**3.903**	**Moderate**
Left thalamus	**3.330**	**Moderate**
Right MGN	**3.021**	**Moderate**
Left AV	**3.008**	**Moderate**
Amygdala		
None	< 3.000	None
IRS		
Hippocampus		
None	< 3.000	None
Thalamus		
Right MV-re	**5.741**	**Moderate**
Left CeM	**4.226**	**Moderate**
Right CeM	**3.352**	**Moderate**
Amygdala		
None	< 3.000	None
CTQ		
Hippocampus		
None	< 3.000	None
Thalamus		
Right CeM	**331.544**	**Extreme**
Left CeM	**330.044**	**Extreme**
Left MV-re	**125.938**	**Extreme**
Left VAmc	**67.682**	**Very strong**
Left Pc	**54.217**	**Very strong**
Left thalamus	**16.651**	**Strong**
Right MV-re	**14.815**	**Strong**
Left VA	**14.370**	**Strong**
Left Pt	**7.336**	**Moderate**
Right VAmc	**5.308**	**Moderate**
Left MGN	**4.536**	**Moderate**
Right thalamus	**4.019**	**Moderate**
Left AV	**3.675**	**Moderate**
Amygdala		
None	< 3.000	None
BAI		
None	< 3.000	None
Age of onset		
None	< 3.000	None

Bold indicates moderate or greater evidence

*MDD* major depressive disorder, *HC* healthy control, *Pt* paratenial nuclear, *L-SG*  limitans-suprageniculate nuclear, *MGN* medical geniculate nuclear, *AV* anteroventral nuclear, *MV-re* medial ventral reuniens nuclear, *CeM* central medial nuclear, *VAmc* ventral anterior magnocellular nuclear, *Pc*  paracentral nuclear, *VA* ventral anterior nuclear

Moreover, moderate evidence was found for higher IRS scores relating to lower volumes within three thalamic subfields (right MV-re: BF_incl_ = 5.741, left CeM: BF_incl_ = 4.226, and right CeM: BF_incl_ = 3.352) in MDD (Table 3, Fig. 3. F–H).

Finally, moderate to extreme evidence was found for more severe childhood trauma relating to lower volumes of bilateral whole thalamus (left: BF_incl_ = 16.651, right: BF_incl_ = 4.019, Table 3, Fig. 3. I&J) and 11 thalamic subfields (right CeM: BF_incl_ = 331.544, left CeM: BF_incl_ = 330.044, left MV-re: BF_incl_ = 125.938, left VAmc: BF_incl_ = 67.682, left Pc: BF_incl_ = 54.217, right MV-re: BF_incl_ = 14.815, left VA: BF_incl_ = 14.370, left Pt: BF_incl_ = 7.336, right VAmc: BF_incl_ = 5.308, left MGN: BF_incl_ = 4.536, and left AV: BF_incl_ = 3.675) in MDD (Table 3, Fig. 3. K-U). No conclusive evidence was found for BAI scores and age of onset (all BF_incl_ < 3).

**Fig. 4:**
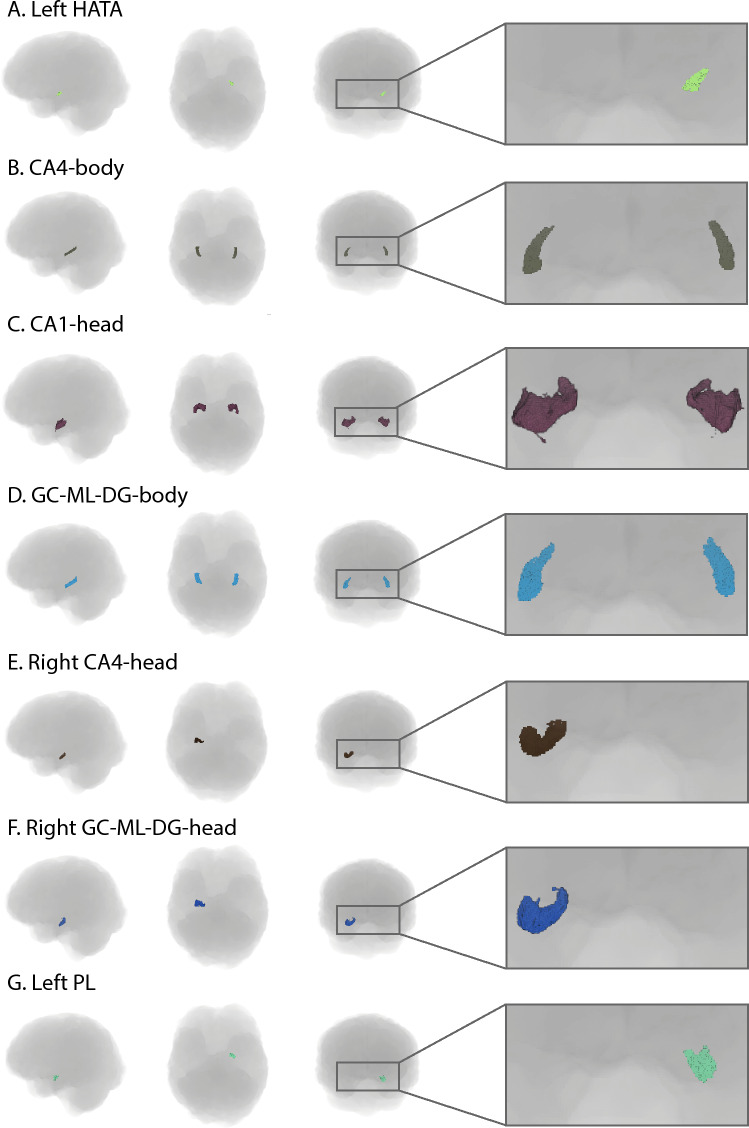
Hippocampal and amygdala subfields with moderate or higher strength evidence in this study. *HATA* hippocampal-amygdala transition area, *CA* cornu Ammonis, *GC-ML-DG* granule cell and molecular layer of the dentate gyrus, *PL* paralaminar nuclear

**Fig. 5:**
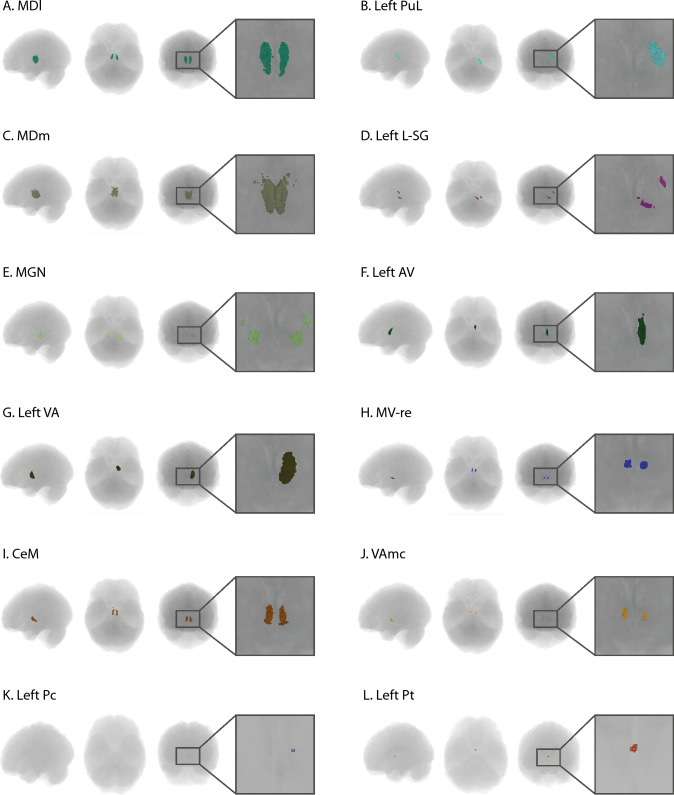
Thalamic subfields with moderate or higher strength evidence in this study. *MDl* mediodorsal lateral parvocellular, *PuL* pulvinar lateral, *MDm* mediodorsal medial magnocellular, *L-SG* limitans-suprageniculate nuclear, *MGN* medical geniculate nuclear, *AV* anteroventral nuclear, *VA* ventral anterior nuclear, *MV-re* medial ventral reuniens nuclear, *CeM* central medial nuclear, *VAmc* ventral anterior magnocellular nuclear, *Pc* paracentral nuclear, *Pt* paratenial nuclear

Figures 4 and 5 show the subfields with moderate or higher strength evidence in this study.

## Discussion

We used UHF MRI to investigate hippocampal, amygdala, and thalamus subfield volumetrics in MDD. While no effect was found for MDD diagnosis (i.e., case–control comparison), clinical characteristics of MDD patients were associated with subfield volumes of the hippocampus, thalamus, and amygdala. Specifically, MDD patients with typical depression versus those with atypical depression showed lower hippocampal, thalamic, and amygdala subfield volumes, with recurrent MDD patients also showing lower thalamic volumes. Finally, symptom severity, childhood trauma, and insomnia across all MDD patients related to lower volumes of various thalamic subfields. These findings allow uniquely fine-grained insights into the morphometry of hippocampal, thalamic, and amygdala subfields, linking some of them to the clinical manifestation of MDD.

### No differences in hippocampal, thalamic, and amygdala subfield volumes between MDD and HC

We did not find conclusive evidence for differences in hippocampal, thalamic, and amygdala subfield volumes in MDD patients compared to HC. Large-scale studies based on 1.5/3.0 Tesla MRI have shown that MDD patients have smaller hippocampal volume compared with HCs [3, 6, 7]. However, several studies using 7.0 Tesla MRI to explore hippocampal subfield volumes between MDD patients and HCs found no significant differences [36, 37, 52], which are consistent with our results. Only one 7.0 Tesla study showed that MDD patients had smaller hippocampal volumes than HCs [53]. These inconsistent results may be the result of different MDD subtypes that lead to clinical heterogeneity between studies [54]. Regarding the amygdala, one study using 7.0 Tesla showed no difference in amygdala subfield volumes in MDD patients compared with HCs [37]. Moreover, as the first study to explore the differences in thalamic subnucleus volumes between patients with MDD and HCs using 7.0 Tesla MRI, we did not find any differences. In this study, we found that MDD diagnosis did not affect subfield volumes compared to the HC. However, within the MDD group, we found that MDD clinical features were associated with the subfield volumes. In addition, we did not find any differences in subfield volumes when all MDD subgroups were compared to HC. In other words, the subfield volumes were moderated by clinical variables but with the absence of a group effect (MDD/HC). This suggests that these subfield volumes are more suitable for differentiating MDD subtypes rather than MDD from HC. The reason is likely that heterogeneity within the MDD group plays a role here. These results prompt us once again to reconsider in what aspects the brain abnormalities in MDD truly manifest.

A previous meta-analysis suggested that the gyrus rectus exhibits the largest volume difference and the largest effect size (-0.72) among various brain regions between MDD patients and HCs, although these effect sizes are presumably artificially high due to publication bias [6]. Though meta-analysis based on consortium data rather than publications also highlights that the largest effect sizes are in the medial orbitofrontal cortex (including the gyrus rectus), even though those were considerably smaller (-0.13) [7, 55]. Additionally, studies utilizing 7.0 Tesla MRI have indicated abnormal white matter structural connections in subfields of the hippocampus and amygdala in MDD patients [56, 57]. Therefore, we speculate that the largest differences in brain volume between individuals with MDD and HCs may not necessarily be in the hippocampus, thalamus, and amygdala but are more likely to be in other brain regions. Additionally, MDD may be more associated with alterations in connectivity in which the thalamus seems to have a primary role [58].

### MDD typical subtype relates to volumes of hippocampal, thalamic, and amygdala subfields

Our study findings suggest that patients with typical MDD had smaller volumes within nine hippocampal subfields (left HATA, right CA4-body, left CA1-head, right GC-ML-DG-body, right CA4-head, right CA1-head, left GC-ML-DG-body, left CA4-body, and right GC-ML-DG-head), five thalamic subfields (right MDl, left MDl, left PuL, right MDm, and left MDm), and one amygdala subfield (left PL), compared with atypical MDD patients.

The hippocampus and its subfields have received widespread attention in the onset and development of depression [3, 59]. Differences in the volumes of the hippocampal CA, ML, and fissure may coincide with or emerge following the onset of depressive symptoms, especially in cases characterized by a chronic course [60]. The function of the hippocampal subfields in this discovery is mainly involved in emotional control and cognitive functions [61, 62]. Inconsistent with our findings, previous evidence suggested that there were no differences in hippocampal volumes between patients with typical and atypical MDD, although a non-statistical trend toward increased hippocampal volume was found in patients with atypical MDD during follow-up [54]. However, the participants included in the previous study were 60 or older, which is substantially different from the age of our included participants.

The function of the thalamic subfields in our result is mainly involved in emotion and memory [38], while the function of the amygdala subfields is mainly involved in emotion and fear learning [63]. Although there are currently no studies reporting on the role of the thalamus and amygdala between typical and atypical depression, several studies reported their association with other subtypes of depression. A previous study showed right fronto-thalamic functional hypoconnectivity compared with HC only in adult-onset MDD but not in early-onset MDD [64]. Using machine learning methods, a study found that the thalamus was an important brain region that distinguishes MDD subtypes [65]. Another study showed that compared with HC, patients with melancholic MDD had increased bilateral thalamic global functional connectivity, but this phenomenon did not exist in patients with non-melancholic MDD [66]. Moreover, patients with anxious MDD had reduced functional connectivity between the right amygdala and right middle frontal gyrus compared with patients with non-anxious MDD [67]. Our study suggests that the volume of hippocampal, thalamic, and amygdala subfields is involved in the pathogenesis of typical/atypical MDD.

### MDD chronicity relates to thalamic subfield volumes

We found that patients with recurrent MDD had smaller left Pc volume compared with patients with first-episode MDD. The Pc is associated with the regulation of attention and arousal [38]. Previous research indicated that patients with recurrent episodes of MDD exhibited reduced functional connectivity between the striatal subfields and the right thalamus when compared to patients with the first episode of MDD [68]. There are currently no studies reporting the role of Pc in first-episode/recurrent MDD, but a study pointed out that the Pc volume in MDD patients was smaller than that in HCs, and was negatively correlated with depressive symptoms [30]. Here, evidence that MDD chronicity relates to volumes of Pc was revealed through detailed segmentation of UHF MRI.

### MDD depressive symptoms, insomnia, and childhood trauma relate to thalamic subnuclei

In the current study, we found that the severity of depressive symptoms, insomnia, and childhood trauma are all negatively associated with the volumes of several thalamic subfields. The thalamic subnuclei involved in this part of the results are mainly related to stress, anxiety, supporting arousal and awareness, and episodic memory [38]. It has been established that anomalies in the thalamus and its subfields are closely related to MDD clinical features [6, 20–22, 30]. Previous evidence showed that the severity of depressive symptoms is inversely related to thalamus volume [30, 69], which is consistent with our findings. Furthermore, the thalamus receives numerous incoming projections from areas that play a role in controlling the sleep––wake cycle [70]. Insomnia is closely associated with the thalamus and its structural/functional connections [71]. A previous study indicated that patients experiencing insomnia exhibit smaller thalamic volumes compared to HCs [72]. Additionally, patients with insomnia display abnormal functional connections between the thalamus and certain brain regions, with these alterations correlating with the severity of insomnia [72]. Moreover, in people with insomnia, there is a reduction in white matter integrity between the thalamus and frontal lobes [73]. Our results reveal an inverse correlation between thalamic subnuclei volumes and insomnia severity in MDD patients.

To our knowledge, the relationship between childhood trauma and thalamus volume in MDD has not been reported previously. Nonetheless, many studies suggest the thalamus plays an important role in the link between other mental illnesses and childhood trauma. In obsessive compulsive disorder, patients with high levels of childhood trauma have increased thalamic resting-state functional connectivity with the prefrontal cortex when compared with HCs [74]. In posttraumatic stress disorder, childhood trauma severity is inversely correlated with the volume of 11 bilateral thalamic subnuclei (VPL, VLa, VLp, VA, VAmc, MDm, Pt, Pc, CeM, CM, and PuA) [75]. Moreover, volume reduction in the thalamus is associated with high levels of childhood trauma in patients with bipolar disorder [76]. Childhood trauma is closely related to the onset and treatment outcome of depression [77–79]. Combined with our findings, there is reason to believe that childhood trauma is closely related to thalamic subnuclei volumes in MDD.

In addition to the effects found for subfield volumes, analysis of volumes of the entire structures showed a lower volume of the right hippocampus in typical compared to atypical MDD participants. Lower volume of the left thalamus was associated with more severe childhood trauma and depressive symptoms. Moreover, a lower volume of the right thalamus was associated with more severe childhood trauma. The volumetric effects in all hippocampal subfields were stronger than those for the entire hippocampus. Those volumetric effects in the entire structure could be strongly driven by specific subfield effects. However, for the severity of depressive symptoms and childhood trauma, the effects of some subfield volumes were weaker than those of the overall volume. These subfield effects do not seem subfield-specific and are more likely related to volumetric effects across the entire structure. The high spatial resolution of UHF MRI leads to a finer subfield segmentation [31–34], which in part helps us distinguish subfield effects from overall effects.

### Strengths and limitations

A particular strength of our study is that we used approximately 3 × higher spatial resolution compared to the current standards (0.7^3^ mm^3^ versus 1.0^3^ mm^3^) and that we included a relatively large number of MDD patients (n = 56), which allowed us to explore the relationship between clinical features and subfield volumes. Due to the financial burden and time constraints at play when conducting UHF MRI research, we chose to maximize and prioritize the inclusion of our main target group (MDD). This resulted in a relatively small HC sample, which not only limits the power to detect subtle differences between MDD and HC, but may also inflate effect sizes. In addition, this is a cross-sectional study, and causal relationships cannot be determined.

## Conclusions

In summary, by utilizing the power of UHF MRI at 7.0 Tesla, we explored the role of hippocampal, thalamic, and amygdala subfield volumes in MDD. Diminished volumes of hippocampal, thalamic, and amygdala subfields seem tightly coupled to typical versus atypical MDD subtype, with thalamic subfield volumes also relating to MDD chronicity. Additionally, diminished volumes of thalamic subfields are associated with depressive symptoms, insomnia, and childhood trauma in MDD patients. These findings enrich our understanding of the neuroanatomy of MDD. Future UHF MRI work should examine how the structural volumes of these subfields change with MDD progression and response to treatment.

## Supplementary Information

Below is the link to the electronic supplementary material.

Supplementary file1 (DOCX 81 KB)

## Data Availability

Data will be available from the corresponding author upon reasonable request.
