# Supplementary material for: Hippocampal, thalamic, and amygdala subfield morphology in major depressive disorder: an ultra-high resolution MRI study at 7-Tesla
Source: Eur Arch Psychiatry Clin Neurosci. 2024 Aug 31;275(4):1113–29. doi: 10.1007/s00406-024-01874-0 (PMC12149001; doi:10.1007/s00406-024-01874-0)
Supplement: Supplementary file 1 — Supplementary file1 (DOCX 81 KB) [file 406_2024_1874_MOESM1_ESM.docx]

| **Supplemental Table 1. Volumes of hippocampal subfields (mm^3^) for MDD vs HC.** | | | | | | |
| --- | --- | --- | --- | --- | --- | --- |
|  | **Left hemisphere** | |  | **Right hemisphere** | |  |
| **Subfields** | **MDD  (n = 56)** | **HC  (n = 14)** | **BF_incl_** | **MDD  (n = 56)** | **HC  (n = 14)** | **BF_incl_** |
| **PARA** | 59152 ± 14749 | 64058 ± 17264 | 0.428 | 63787 ± 12077 | 63633 ± 18351 | 0.304 |
| **PSIL-head** | 121434 ± 31569 | 131116 ± 28180 | 0.422 | 131159 ± 25209 | 127078 ± 38089 | 0.314 |
| **PSIL-body** | 143360 ± 42251 | 152517 ± 57891 | 0.331 | 151063 ± 28901 | 148366 ± 45254 | 0.313 |
| **SIL-head** | 177997 ± 47097 | 189423 ± 47662 | 0.331 | 187631 ± 41368 | 178381 ± 53653 | 0.366 |
| **SIL-body** | 229002 ± 57173 | 234080 ± 71593 | 0.298 | 232181 ± 42640 | 224476 ± 72744 | 0.314 |
| **CA1-head** | 527551 ± 133048 | 568707 ± 132818 | 0.359 | 574592 ± 102885 | 582866 ± 190710 | 0.302 |
| **CA1-body** | 134038 ± 39462 | 136814 ± 27681 | 0.296 | 147525 ± 23037 | 144746 ± 45271 | 0.315 |
| **CA3-head** | 105453 ± 24583 | 115577 ± 30151 | 0.504 | 117465 ± 20460 | 127026 ± 42508 | 0.523 |
| **CA3-body** | 71238 ± 22141 | 71284 ± 15039 | 0.299 | 86156 ± 19773 | 87120 ± 26651 | 0.298 |
| **CA4-head** | 125000 ± 30023 | 137030 ± 35355 | 0.474 | 137294 ± 23898 | 145587 ± 50923 | 0.383 |
| **CA4-body** | 113112 ± 28030 | 119699 ± 28495 | 0.319 | 118240 ± 18829 | 116849 ± 37627 | 0.330 |
| **GC-ML-DG-head** | 147899 ± 34878 | 161998 ± 40337 | 0.477 | 163315 ± 27524 | 172051 ± 57481 | 0.371 |
| **GC-ML-DG-body** | 123528 ± 30376 | 131685 ± 32131 | 0.328 | 127561 ± 19470 | 125325 ± 37470 | 0.348 |
| **ML-head** | 107877 ± 31714 | 118856 ± 27336 | 0.425 | 122323 ± 32159 | 125624 ± 37326 | 0.305 |
| **ML-body** | 102381 ± 34397 | 116085 ± 32627 | 0.460 | 98538 ± 26199 | 93250 ± 21498 | 0.375 |
| **HATA** | 53222 ± 14361 | 58251 ± 18999 | 0.453 | 61857 ± 14615 | 61794 ± 23530 | 0.294 |
| **Fimbria** | 77394 ± 17467 | 88567 ± 28033 | 0.800 | 74333 ± 20487 | 74441 ± 30143 | 0.305 |
| **Tail** | 491367 ± 121457 | 480765 ± 118007 | 0.324 | 551519 ± 89669 | 532529 ± 175203 | 0.347 |
| **Fissure** | 174421 ± 49933 | 174227 ± 56341 | 0.296 | 192727 ± 42455 | 180094 ± 63448 | 0.332 |
| **Whole** | 2911006 ± 633633 | 3076513 ± 670665 | 0.331 | 3146538 ± 449380 | 3131141 ± 934979 | 0.295 |
| Abbrivations: PARA = parasubiculum; PSIL = presubiculum; SIL = subiculum; CA = cornu Ammonis; GC-ML-DG = granule cell and molecular layer of the dentate gyrus; ML = molecular layer; HATA = hippocampal-amygdala transition area. | | | | | | |

| **Supplemental Table 2. Volumes of thalamic subnuclei (mm^3^) for MDD vs HC.** | | | | | | |
| --- | --- | --- | --- | --- | --- | --- |
|  | **Left hemisphere** | |  | **Right hemisphere** | |  |
| **Subfields** | **MDD  (n = 56)** | **HC  (n = 14)** | **BF_incl_** | **MDD  (n = 56)** | **HC (n = 14)** | **BF_incl_** |
| **AV** | 129496 ± 20032 | 123530 ± 25122 | 0.493 | 134339 ± 19846 | 130540 ± 20603 | 0.419 |
| **LD** | 28823 ± 8015 | 27307 ± 10391 | 0.342 | 27225 ± 7710 | 23342 ± 6221 | 0.874 |
| **LP** | 124626 ± 17710 | 129503 ± 26645 | 0.339 | 114018 ± 19278 | 111806 ± 27761 | 0.313 |
| **VA** | 439938 ± 47750 | 428072 ± 59298 | 0.512 | 413649 ± 46585 | 413669 ± 62900 | 0.390 |
| **VAmc** | 32761 ± 4074 | 31606 ± 3909 | 0.585 | 32233 ± 3568 | 31331 ± 4393 | 0.535 |
| **VLa** | 621262 ± 61406 | 619038 ± 86405 | 0.354 | 603493 ± 62013 | 605439 ± 92163 | 0.350 |
| **VLp** | 794636 ± 77797 | 808338 ± 120065 | 0.302 | 766033 ± 74708 | 776309 ± 127143 | 0.311 |
| **VPL** | 847788 ± 96009 | 881933 ± 121810 | 0.336 | 796490 ± 75827 | 814147 ± 113809 | 0.301 |
| **VM** | 18222 ± 2049 | 18928 ± 2647 | 0.337 | 17519 ± 1893 | 17975 ± 2436 | 0.302 |
| **CM** | 237670 ± 26185 | 240847 ± 29413 | 0.309 | 229780 ± 21798 | 230821 ± 35243 | 0.317 |
| **CeM** | 66296 ± 11705 | 62338 ± 13181 | 0.650 | 65861 ± 10533 | 61672 ± 13002 | 0.873 |
| **CL** | 33328 ± 7316 | 32257 ± 7815 | 0.329 | 31431 ± 5899 | 28105 ± 6535 | 1.089 |
| **Pc** | 3980 ± 0.484 | 3856 ± 0.440 | 0.474 | 4406 ± 0.510 | 4242 ± 0.634 | 0.641 |
| **Pf** | 50751 ± 6282 | 52966 ± 10848 | 0.397 | 51873 ± 6061 | 52449 ± 11200 | 0.299 |
| **Pt** | 6471 ± 0.763 | 6584 ± 0.957 | 0.311 | 6760 ± 0.618 | 6795 ± 0.985 | 0.304 |
| **MV-re** | 12625 ± 3321 | 11438 ± 4141 | 0.648 | 13027 ± 3410 | 11420 ± 4154 | 1.120 |
| **MDm** | 835329 ± 71126 | 816510 ± 83736 | 0.562 | 833374 ± 71945 | 837771 ± 102865 | 0.320 |
| **MDI** | 300902 ± 25287 | 292198 ± 31742 | 0.532 | 297259 ± 28023 | 303506 ± 44070 | 0.304 |
| **LGN** | 294024 ± 37642 | 293925 ± 35136 | 0.335 | 266258 ± 39560 | 254308 ± 46643 | 0.535 |
| **MGN** | 117394 ± 16024 | 120909 ± 20406 | 0.307 | 111852 ± 16334 | 108312 ± 17026 | 0.486 |
| **L-SG** | 33213 ± 6604 | 34137 ± 7821 | 0.306 | 27171 ± 6433 | 26171 ± 5860 | 0.341 |
| **PuA** | 219797 ± 24916 | 223123 ± 36414 | 0.314 | 237972 ± 26185 | 244297 ± 34497 | 0.303 |
| **PuM** | 1071655 ± 125019 | 1109024 ± 170684 | 0.299 | 1240616 ± 119199 | 1274629 ± 175796 | 0.306 |
| **PuL** | 164016 ± 35045 | 170880 ± 47721 | 0.300 | 209661 ± 38664 | 223712 ± 43092 | 0.357 |
| **PuI** | 240972 ± 38262 | 245258 ± 41935 | 0.293 | 297902 ± 35901 | 305444 ± 38971 | 0.327 |
| **Whole** | 6725976 ± 574986 | 6784505 ± 848512 | 0.329 | 6830201 ± 548261 | 6898212 ± 903865 | 0.325 |
| Abbrivations: AV = anteroventral nucleus; LD = laterodorsal nucleus; LP = lateral posterior nucleus; VA = ventral anterior nucleus; VAmc = ventral anterior magnocellular nucleus; Vla = ventral lateral anterior nucleus; VLp = ventral lateral posterior nucleus; VPL = ventral posterolateral nucleus; VM = ventromedial nucleus; CM = centromedian nucleus; CeM = central medial nucleus; CL = central lateral nucleus; Pc = paracentral nucleus; Pf = parafascicular nucleus; Pt = paratenial nucleus; MV-re = medial ventral reuniens nucleus; MDm = mediodorsal medial magnocellular nucleus; MDI = mediodorsal lateral parvocellular nucleus; LGN = lateral geniculate nucleus; MGN = medical geniculate nucleus; L-SG = limitans-suprageniculate nucleus; PuA = pulvinar anterior nucleus; PuM = pulvinar medial nucleus; PuL = pulvinar lateral nucleus; PuI = pulvinar inferior nucleus. | | | | | | |

| **Supplemental Table 3. Volumes of amygdala subfields (mm^3^) for MDD vs HC.** | | | | | | |
| --- | --- | --- | --- | --- | --- | --- |
|  | **Left hemisphere** | |  | **Right hemisphere** | |  |
| **Subfields** | **MDD  (n = 56)** | **HC  (n = 14)** | **BF_incl_** | **MDD  (n = 56)** | **HC  (n = 14)** | **BF_incl_** |
| **La** | 581956 ± 114858 | 629971 ± 122679 | 0.518 | 629052 ± 79483 | 625951 ± 179416 | 0.310 |
| **Ba** | 396910 ± 86623 | 423336 ± 105366 | 0.363 | 436705 ± 70490 | 428443 ± 136649 | 0.322 |
| **AB** | 239461 ± 59465 | 253540 ± 74210 | 0.356 | 272334 ± 51701 | 263580 ± 87097 | 0.340 |
| **Ce** | 43977 ± 13406 | 45894 ± 16934 | 0.348 | 51293 ± 10981 | 47808 ± 15592 | 0.504 |
| **Me** | 25174 ± 9419 | 25298 ± 10194 | 0.302 | 27044 ± 7490 | 23579 ± 9207 | 0.640 |
| **Co** | 24652 ± 7482 | 25258 ± 8370 | 0.313 | 28010 ± 6030 | 26466 ± 9099 | 0.356 |
| **PL** | 46185 ± 10418 | 49407 ± 11940 | 0.392 | 48193 ± 8180 | 46225 ± 14950 | 0.389 |
| **CAT** | 176740 ± 40362 | 187739 ± 49081 | 0.364 | 191731 ± 35499 | 185460 ± 62887 | 0.339 |
| **AAA** | 49251 ± 10401 | 50481 ± 12537 | 0.307 | 54917 ± 9269 | 51592 ± 17033 | 0.411 |
| **Whole** | 1584306 ± 331742 | 1690924 ± 387419 | 0.380 | 1739278 ± 256848 | 1699104 ± 524658 | 0.339 |
| Abbrivations: La = lateral nucleus; Ba = basal nucleus; AB = accessory basal nucleus; Ce = central nucleus; Me = medial nucleus; Co = cortical nucleus; PL = paralaminar nucleus; CAT = cortico-amngdaloid transition area; AAA = anterior amygdala area. | | | | | | |

| **Subplemental Table 4. Summary of the BF values of subfield volumes for subgroups versus HC.** | | | | | |
| --- | --- | --- | --- | --- | --- |
|  | |  | |  | **BF_incl_** |
| **Typical MDD versus HC** |  |  |  |  |  |
| None |  |  |  |  | < 3.000 |
|  |  |  |  |  |  |
| **Atypical MDD versus HC** |  |  |  |  |  |
| None |  |  |  |  | < 3.000 |
|  |  |  |  |  |  |
| **First episode MDD versus HC** |  |  |  |  |  |
| None |  |  |  |  | < 3.000 |
|  |  |  |  |  |  |
| **Recurrent MDD versus HC** |  |  |  |  |  |
| None |  |  |  |  | < 3.000 |
|  |  |  |  |  |  |
| **Medicated MDD versus HC** |  |  |  |  |  |
| None |  |  |  |  | < 3.000 |
|  |  |  |  |  |  |
| **Unmedicated MDD versus HC** |  |  |  |  |  |
| None |  |  |  |  | < 3.000 |
|  | | | | |  |
| Abbreviation: MDD = major depressive disorder; HC = healthy control. | | | | | |

| **Subplemental Table 5. Demographical and clincal information of the typical and atypical MDD participants.** | | | | | | | | | |
| --- | --- | --- | --- | --- | --- | --- | --- | --- | --- |
|  | | **Typical MDD**  **(n = 41)** | **Atypical MDD**  **(n = 14)** | | | **BF_10_** | | **Strength of**  **evidence** | |
| Age (years), mean ± SD |  | 37.87 ± 10.33 | |  | 33.20 ± 10.86 | 0.693 | | None | |
| Gender (female), n (%) |  | 30 (73.17%) | |  | 11 (78.57%) | 0.336 | | None | |
| Age of onset (years) ^a^, mean ± SD |  | 21.78 ± 11.98 | |  | 20.57 ± 6.64 | 0.320 | | None | |
| Recurrent depression, n (%) |  | 18 (43.90%) | |  | 5 (35.71%) | 0.410 | | None | |
| IDS, mean ± SD |  | 30.76 ± 13.41 | |  | 43.71 ± 7.91 | **25.054** | | **Strong** | |
| CTQ, mean ± SD |  | 47.32 ± 17.48 | |  | 47.14 ± 18.65 | 0.304 | | None | |
| BAI, mean ± SD |  | 12.44 ± 9.28 | |  | 19.07 ± 9.02 | 2.517 | | None | |
| IRS, mean ± SD |  | 9.44 ± 5.27 | |  | 11.71 ± 4.78 | 0.680 | | None | |
| With any psychotropic medications, n (%) |  | 20 (48.78%) | |  | 12 (85.71%) | **6.774** | | **Moderate** | |
| With any antidepressants, n (%) |  | 19 (46.34%) | |  | 10 (71.43%) | 1.292 | | None | |
| With SSRIs and/or SNRIs, n (%) |  | 15 (36.59%) | |  | 7 (50.00%) | 0.531 | | None | |
| With TCA, n (%) |  | 5 (12.20%) | |  | 3 (21.43%) | 0.397 | | None | |
| With atypical antidepressants, n (%) |  | 1 (2.44%) | |  | 1 (7.14%) | 0.230 | | None | |
| With lithium stabilizer, n (%) |  | 2 (4.88%) | |  | 1 (7.14%) | 0.203 | | None | |
| With antipsychotics, n (%) |  | 3 (7.32%) | |  | 3 (21.43%) | 0.664 | | None | |
| With benzodiazepines, n (%) |  | 5 (12.20%) | |  | 2 (14.29%) | 0.265 | | None | |
|  | | | | | | |  | |  |
| Abbreviation: MDD = major depressive disorder; HC = healthy control; SD = standard deviation; IDS = Inventory for Depressive Symptomatology; CTQ = Childhood Trauma Questionnaire; BAI = Becks Anxiety Inventory; IRS = Insomnia Rating Scale; SSRI = selective serotonin reuptake inhibitor; SNRI = serotonin-norepinephrine reuptake inhibitor; TCA = tricyclic antidepressant.  ^a^: One typical MDD patient did not provide the age of onset. Therefore, when analyzing the data related to age of onset, the sample size of typical MDD patients is 40 individuals. | | | | | | | | | |
|  | | | | | | |  | |  |
|  | | | | | | |  | |  |
|  | | | | | | |  | |  |
|  | | | | | | |  | |  |
|  | | | | | | |  | |  |

| **Subplemental Table 6. Demographical and clincal information of the first episode and recurrent MDD participants.** | | | | | | | | | |
| --- | --- | --- | --- | --- | --- | --- | --- | --- | --- |
|  | | **First episode MDD**  **(n = 32)** | **Recurrent  MDD**  **(n = 23)** | | | **BF_10_** | | **Strength of**  **evidence** | |
| Age (years), mean ± SD |  | 35.43 ± 10.06 | |  | 38.42 ± 11.24 | 0.428 | | None | |
| Gender (female), n (%) |  | 24 (75.00%) | |  | 17 (73.91%) | 0.290 | | None | |
| Age of onset (years) ^a^, mean ± SD |  | 21.42 ± 9.73 | |  | 21.52 ± 12.33 | 0.277 | | None | |
| With atypical MDD ^a^, n (%) |  | 9 (28.13%) | |  | 5 (21.74%) | 0.326 | | None | |
| IDS, mean ± SD |  | 34.75 ± 13.58 | |  | 33.09 ± 13.49 | 0.299 | | None | |
| CTQ, mean ± SD |  | 47.81 ± 16.96 | |  | 46.52 ± 18.84 | 0.283 | | None | |
| BAI, mean ± SD |  | 12.72 ± 9.38 | |  | 16.09 ± 9.72 | 0.548 | | None | |
| IRS, mean ± SD |  | 9.97 ± 5.63 | |  | 10.09 ± 4.68 | 0.276 | | None | |
| With any psychotropic medications, n (%) |  | 19 (59.38%) | |  | 13 (56.52%) | 0.332 | | None | |
| With any antidepressants, n (%) |  | 18 (56.25%) | |  | 11 (47.83%) | 0.395 | | None | |
| With SSRIs and/or SNRIs, n (%) |  | 11 (34.38%) | |  | 11 (47.83%) | 0.527 | | None | |
| With TCA, n (%) |  | 6 (18.75%) | |  | 2 (8.70%) | 0.376 | | None | |
| With atypical antidepressants, n (%) |  | 1 (3.13%) | |  | 1 (4.35%) | 0.143 | | None | |
| With lithium stabilizer, n (%) |  | 3 (9.38%) | |  | 0 (0.00%) | 0.374 | | None | |
| With antipsychotics, n (%) |  | 1 (3.13%) | |  | 5 (21.74%) | 1.904 | | None | |
| With benzodiazepines, n (%) |  | 3 (9.38%) | |  | 4 (17.39%) | 0.327 | | None | |
|  | | | | | | |  | |  |
| Abbreviation: MDD = major depressive disorder; HC = healthy control; SD = standard deviation; IDS = Inventory for Depressive Symptomatology; CTQ = Childhood Trauma Questionnaire; BAI = Becks Anxiety Inventory; IRS = Insomnia Rating Scale; SSRI = selective serotonin reuptake inhibitor; SNRI = serotonin-norepinephrine reuptake inhibitor; TCA = tricyclic antidepressant.  ^a^: One first episode MDD patient did not provide the age of onset. Therefore, when analyzing the data related to age of onset, the sample size of first episode MDD patients is 31 individuals. | | | | | | | | | |
|  | | | | | | |  | |  |
|  | | | | | | |  | |  |
|  | | | | | | |  | |  |
|  | | | | | | |  | |  |
|  | | | | | | |  | |  |

| **Subplemental Table 7. Demographical and clincal information of the medicated and unmedicated MDD participants.** | | | | | | | | |
| --- | --- | --- | --- | --- | --- | --- | --- | --- |
|  | | **Non-medicated MDD**  **(n = 23)** | **Medicated  MDD**  **(n = 32)** | | **BF_10_** | | **Strength of**  **evidence** | |
| Age (years), mean ± SD |  | 36.22 ± 11.10 |  | 37.02 ± 10.34 | 0.284 | | None | |
| Gender (female), n (%) |  | 15 (65.22%) |  | 26 (81.25%) | 0.693 | | None | |
| Age of onset (years) ^a^, mean ± SD |  | 22.14 ± 11.67 |  | 21.00 ± 10.34 | 0.295 | | None | |
| Recurrent depression, n (%) |  | 10 (43.48%) |  | 13 (40.63%) | 0.332 | | None | |
| With atypical MDD, n (%) |  | 2 (8.70%) |  | 12 (37.50%) | **5.389** | | **Moderate** | |
| IDS, mean ± SD |  | 27.96 ± 13.67 |  | 38.44 ± 11.61 | **11.281** | | **Strong** | |
| CTQ, mean ± SD |  | 44.74 ± 14.98 |  | 49.09 ± 19.31 | 0.385 | | None | |
| BAI, mean ± SD |  | 8.96 ± 5.94 |  | 17.84 ± 10.04 | **68.832** | | **Very strong** | |
| IRS, mean ± SD |  | 9.57 ± 5.32 |  | 10.34 ± 5.18 | 0.311 | | None | |
|  | | | | | |  | |  |
| Abbreviation: MDD = major depressive disorder; HC = healthy control; SD = standard deviation; IDS = Inventory for Depressive Symptomatology; CTQ = Childhood Trauma Questionnaire; BAI = Becks Anxiety Inventory; IRS = Insomnia Rating Scale; SSRI = selective serotonin reuptake inhibitor; SNRI = serotonin-norepinephrine reuptake inhibitor; TCA = tricyclic antidepressant.  ^a^: One non-medicated MDD patient did not provide the age of onset. Therefore, when analyzing the data related to age of onset, the sample size of non-medicated MDD patients is 22 individuals. | | | | | | | | |
|  | | | | | |  | |  |
|  | | | | | |  | |  |
|  | | | | | |  | |  |
|  | | | | | |  | |  |
|  | | | | | |  | |  |
